# A New Differential Diagnosis: Synthetic Cannabinoid-Associated Gross Hematuria

**DOI:** 10.1155/2019/6327819

**Published:** 2019-02-24

**Authors:** Osamah Hasan, Ankit A. Patel, James J. Siegert

**Affiliations:** ^1^Midwestern University, 555 31st St., Downers Grove, IL 60515, USA; ^2^Franciscan Health, 20201 South Crawford Avenue, Olympia Fields, IL 60461, USA

## Abstract

Recreational use of synthetic cannabinoids (SCs), also known as “K2” or “Spice,” is becoming a major public-health concern due to their potential for abuse and harmful consequences. New substances are constantly being added to the content of SCs. The dearth of information on these newly added contents as they are introduced into the black market hinders risk assessments of these compounds. We report a highly unusual case of gross hematuria in a 28-year-old male patient after SC use. He was found to have a supratherapeutic INR with no history of prior anticoagulation. His hematuria resolved after four units of fresh-frozen plasma were administered. We include a literature review of the clinical effects of SCs and their possible mechanism of gross hematuria and management.

## 1. Case Report

A 28-year-old male presented to the emergency department with a two-day history of epistaxis and one-day duration of painless gross hematuria. He described his urine initially “fruit punch-like” with progression to “somewhat like ketchup” prior to presentation. He denied dysuria, frequency, urgency, incomplete bladder emptying, clot formation, and flank or abdominal pain. The patient denied any previous episodes of hematuria, history of nephrolithiasis, smoking tobacco, genitourinary malignancies, or any kidney issues in the past. On presentation, the patient was found to have an International Normalised Ratio (INR) greater than 11 despite no prior history of anticoagulation or hepatic dysfunction. Serum laboratory findings were significant for hemoglobin of 13.3, white blood count of 18.9 × 10^3^/*μ*L, and creatinine of 0.9 mg/dL (Table 1). Urinalysis demonstrated >100 RBCs and >100 WBCs. CT urogram with IV-infused iodine contrast solution was negative for upper tract pathology. Urine cultures taken at the time of admission was contaminated, and no additional cultures were taken. Further discussion with the patient revealed that the patient smoked Spice intermittently, and the last smoking episode was 24 hours ago. The SC specimen was not available for evaluation. His INR downtrended to 2.9 after four units of fresh-frozen plasma were administered, and his gross hematuria resolved by the time of discharge a day later with INR of 1.9. The patient was encouraged to follow-up for a cystoscopy in three days; however, he was lost to follow-up and his hematuria and coagulopathy could not be further assessed.

## 2. Discussion

Synthetic cannabinoids (SCs) are robust compounds that are a popular alternative to marijuana. SCs were first created in the 1980s for lab use to study human cannabinoid receptor systems but spilled into the US market as “K2” or “Spice” [1, 2]. Tetrahydrocannbinol (THC) compounds present in marijuana are partial agonists of cannabinoid receptors that are responsible for inducing psychoactive properties. SCs differ from marijuana as SCs display full agonistic effects on cannabinoid receptor (CBR) types 1 and 2 [2]. These synthetic THC compounds encompass a mixture of cyclohexyl phenols (CP compounds) and John W. Huffman (JWH) compounds, the byproducts of which are excreted in urine [3]. Recreational drug users quickly identified these synthetic compounds are undetectable on routine urine immunoassays for drug tests and provides a greater “high,” thereby increasing its demand and popularity. Illicit manufacturers continually introduce newer compounds to SCs as toxicology labs trail behind identifying some of these substances [2]. Whether these breakdown products have a direct impact on the genitourinary health has yet to be determined.

Multiple case series have been reported in the literature that show damaging genitourinary outcomes from SC use. Bhanushali et al. reported four cases of acute kidney injury (AKI) after Spice use from the same city. Renal biopsy findings from three patients showed acute tubular necrosis (ATN) [4]. None of the patients required renal placement therapy and simultaneously recovered without specific treatment [4]. Zarifi and Vyas also hypothesized ATN as a mechanism for SC-induced AKI in their patient, who initially presented with seizures [2]. In their systematic review of 35 case reports, they found ATN as the most common mechanism of injury after SC use [2]. Acute interstitial nephritis and calcium oxalate crystals have been found on renal biopsy as well [2]. Symptoms have generally occurred 12–48 hours after Spice use requiring admission [5]. Cannabinoid metabolites XLR-11, AM-2201, and UR-144 have been isolated in patients with K2/Spice-induced AKI [2]. However, to date, no studies have showed the mechanism by which these byproducts might induce renal failure.

While it may be possible that newer byproducts in fresher SC batches can cause ATN, our index patient presented starkly different from the ones who were found to have AKI. Our patient presented with epistaxis and painless gross hematuria with normal renal function. The lack of nausea, vomiting, abdominal pain, or renal failure as seen in other studies [2,4–6] are a novel presentation of post-SC use in the context of a negative workup. In addition, the supratherapeutic INR in our case suggested that an anticoagulant was added in the circulating Spice mixture.

An initial widespread search in medical literature failed to reveal previous reports of SC-induced supratherapeutic INR or painless gross hematuria. However, numerous articles were published recently including one from the CDC that reported 4 deaths and 155 hospitalizations across Illinois from severe unexplained bleeding and supratherapeutic INRs [7]. Gross hematuria was the most common presenting symptom [7]. Kelkar et al. reported intraparenchymal hemorrhage in their patient who presented with GCS of 4 after SC use [8]. The Illinois Department of Public Health quickly responded by developing syndromic queries and case definitions to easily identify these patients [7]. Our patient fits the criteria of a “probable case” of a SC-associated coagulopathy because our patient reported the use of SC prior to presenting with symptoms and lab findings showed elevated INR [7]. Serum tests such as the anticoagulant poisoning panel were not ordered for our patient to identify the specific anticoagulant nor was the SC specimen available for further evaluation.

SCs were found to contain brodifacoum, a rat poison, which prevents blood from clotting [7]. Also known as a “superwarfarin,” brodifacoum has the same mechanism of action as warfarin: reduction of vitamin K-dependent clotting factor synthesis by interruption of the vitamin K epoxide [9]. In addition, serum testing in patients also confirmed the presence of brodifacoum [9]. Brodifacoum has a prolonged half-life because it is stored in lipids and can be released over long periods of time. This is hypothesized to be the rationale behind the intentional lacing of SC with brodifacoum for a theoretical “longer-lasting” effect of the drug [9]. This potentially may explain our patient's unexplained hematuria.

Currently, there are no FDA-approved drugs that eliminate brodifacoum from the blood. However, combinations of IV vitamin K_1_, red-cell transfusion, fresh-frozen plasma transfusion, and 4-factor prothrombin complex concentrate were used to reverse coagulopathy in patients according to their clinical presentations [7–9]. The Illinois Poison Control set forth guidelines to reverse long-acting anticoagulants by discharging the patient with oral 50–100 mg/day vitamin K_1_ for weeks to months to maintain normal INRs [9]. Our patient's INR normalised to 1.9 at the time of discharge and was therefore not provided with oral vitamin K_1_.

## 3. Conclusion

This case report raises new public health awareness of potential hematologic and urologic manifestations of synthetic cannabinoid (SC) use. Due to the lack of controlled trials and variation of different compounds that make up SCs, little is known about the pathologic mechanism of its clinical outcomes, especially gross hematuria. Physicians should now be aware that these conditions likely are attributable to supratherapeutic INR from mixture of rat poison with Spice/K2 SCs. Appropriate treatment protocols should be initiated to reverse coagulopathy and maintain normal INRs. Furthermore, poison control/state health departments should be notified in cases of unexplained symptoms and when SC use is suspected.

## Figures and Tables

**Table 1:** Chem-8 panel and liver functions tests on admission.

Na	K	Cl	HCO_3_	BUN	Cr	Glucose	Ca	AST	ALT
142	3.9	108	28	16	0.9	105	8.6	17	11
